# Myeloid-Derived Suppressor Cells in Colorectal Cancer

**DOI:** 10.3389/fimmu.2020.01526

**Published:** 2020-08-07

**Authors:** Izabela Sieminska, Jarek Baran

**Affiliations:** Department of Clinical Immunology, Jagiellonian University Medical College, Krakow, Poland

**Keywords:** colorectal cancer (CRC), myeloid-derived suppressor cells (MDSCs), inducible NO synthase (iNOS), arginase-1 (ARG1), T regulatory cells (Tregs)

## Abstract

Colorectal cancer (CRC) remains one of the most common malignancies diagnosed worldwide. The pathogenesis of CRC is complex and involves, among others, accumulation of genetic predispositions and epigenetic factors, dietary habits, alterations in gut microbiota, and lack of physical activity. A growing body of evidence suggests that immune cells play different roles in CRC, comprising both pro- and anti-tumorigenic functions. Immunosuppression observed during cancer development and progression is a result of the orchestration of many cell types, including myeloid-derived suppressor cells (MDSCs). MDSCs, along with other cells, stimulate tumor growth, angiogenesis, and formation of metastases. This article focuses on MDSCs in relation to their role in the initiation and progression of CRC. Possible forms of immunotherapies targeting MDSCs in CRC are also discussed.

## Introduction

### Colorectal Cancer (CRC): Epidemiology and Immunity

According to the World Cancer Research Foundation, colorectal cancer (CRC) (referring to malignancy of colon, rectum, or anus) is the third most common malignancy worldwide. In 2018, more than 1.8 million new cases of CRC were diagnosed (1). About 20–25% of CRC cases are caused by genetic predispositions, including monogenic mutations in mismatched repairing genes associated with, e.g., DNA repair, the cell cycle, and apoptosis (2). Alongside inherited genetic mutations, epigenetic changes also play a significant role in CRC development (3). The remaining 75–80% of cases develop spontaneously and are related to environmental factors such as lack of physical activity, dietary habits, and smoking or alcohol abuse (4). Currently, alterations in the composition of the gut microbiome and its metabolites (playing a role in damaging local tolerance) are also considered as risk factors for CRC (5). An increased risk of CRC is often associated with chronic inflammation of the mucous membrane, which may lead to cell dysplasia, as was proven for patients with inflammatory bowel disease (IBD) (6).

The role of inflammation in CRC development was further supported by data showing that non-steroidal anti-inflammatory drugs (NSAIDs) may decrease the risk of both CRC and colon polyps, which are considered as a premalignant stage (7, 8). The tumor-infiltrating leukocytes (TILs), especially lymphocytes, contribute to the immunoscore classification, where the density of CD3^+^ and CD8^+^ T-cell infiltrate is used as a predictor of anti-tumor response and the prognostic marker in CRC (9, 10). However, further studies have shown that most of the immune cells may actually have a dual activity—anti- and pro-tumor, depending on the signals received from the tumor microenvironment. Interestingly, the so-called myeloid-derived suppressor cells (MDSCs) can switch the polarization of other cells to the status with pro-tumorigenic activity (11).

## Myeloid-Derived Suppressor Cells (MDSCs)

Already in the early 1900s, it was shown that cancer development is often accompanied by extra-medullary hematopoiesis (EMH) and neutrophilia (12). These “fresh” leukocytes were further characterized by suppressive activity and were called immature myeloid cells (ImC) or myeloid suppressor cells (MSC) (13). Eventually, in 2007, their name was changed to MDSCs (13). These cells represent a heterogeneous population of granulocytes and monocytes that rapidly expand during infection, inflammation, and cancer (14, 15). MDSCs, together with the tumor-associated neutrophils (TANs), tumor-associated macrophages (TAMs), and regulatory dendritic cells, compose the population of myeloid regulatory cells (MRC), strongly cooperating with each other during cancer development, and progression (16). Based on mouse data, the MDSC population has been divided into two subgroups: of monocyte (Mo-MDSCs), defined as CD11b^+^Ly6G^−^Ly6C^high^, and polymorphonuclear (PMN-MDSCs), CD11b^+^Ly6G^+^Ly6C^low^, origin (11, 17, 18). Reflecting MDSC populations already defined in mice, human MDSCs have been described as Lin^−^ HLA-DR^−^/low CD11b^+^ CD14^−^ CD15^+^ CD33^+^ for PMN-MDSCs and Lin^−^ HLA-DR^−^/low CD11b^+^ CD14^+^ CD15^−^ CD33^+^ for Mo-MDSCs. Very recently, a population of early-stage MDSCs (e-MDSCs) was detected and defined as Lin^−^ HLA-DR^−^/low CD11b^+^ CD14^−^ CD15^−^ CD33^+^ (17, 19). As their name suggests, these cells possess immunosuppressive function and help cancer to escape the surveillance of the immune system and support further tumor development (17). Most studies point out that the suppressive role of MDSCs in cancer is associated with the activation of their two enzymes, namely inducible NO synthase (iNOS) and arginase-1 (ARG1) (20–22). These enzymes are responsible for metabolism of L-arginine, which is essential for the proliferation and proper functioning of T cells (23). Moreover, NO and ROS produced in these reactions are involved in the inactivation of the T-cell receptor (TCR), causing a decrease in the expression of CD3ζ chain and inducing T-cell apoptosis (18, 19, 22).

### Expansion and Activation of MDSCs in CRC

It is widely accepted that the level of circulating MDSCs increases in the late stage of cancer, correlating with disease progression and formation of metastases (15, 24–26). However, recently, Ma et al. showed that the MDSC level in circulation also increases in premalignant states, such as colon polyposis (27).

The development of MDSCs is caused by various mediators released under chronic inflammatory conditions, including the release of chemokines (11, 15, 28, 29). One of them that is particularly relevant is CCL2, which contributes to tumor growth, progression, and metastasis development in many tumors, including breast, ovarian, prostate, and CRC (30–33). Previous studies in mice showed that CRC growth could be supported by myeloid cells recruited by the CCL2-CCR2 signaling pathway (33). CCL2 caused accumulation of MDSCs and enhanced their immunosuppressive function during colorectal carcinogenesis (34). It was also shown that the level of CCL2 increased simultaneously with the progression of CRC (humans), while the deletion of CCL2 led to the reduction of the MDSC level (mouse model) (34). Further, RNS produced by MDSCs may nitrite chemokines, e.g., CCL2 to N-CCL2, which do not attract CD8+ T cells (like unmodified CCL2 does) but instead recruit myeloid cells, e.g., monocytes (35). On the other hand, several studies documented that CXCL1 is elevated in human CRC (36–38). Further data indicated that CXCR2-positive MDSCs are recruited through CXCR2 ligands, e.g., CXCL1 and CXCl2 are essential for chronic colonic inflammation and colitis-associated tumorigenesis (39).

In addition to chemokines, an important role in the regulation of MDSC activity is attributed to other inflammatory mediators such as histamine and prostaglandins. It has been documented that histamine induces MDSC proliferation and promotes ARG1 and iNOS expression in Mo-MDSCs. At the same time, histamine inhibits the expression of ARG1 and iNOS in PMN-MDSCs, promoting the production of IL-13 and IL-4 (40). Thus, histamine may activate Mo-MDSCs and PMN-MDSCs in different ways (40, 41). Prostaglandin E2 (PGE2), on the other hand, is a strong proinflammatory mediator produced by COX-2 (42) and may activate MDSCs through STAT3 phosphorylation (43, 44). In CRC, persistent STAT3 activation is associated with tumor growth (45, 46) and activation of MDSCs (47, 48). These observations are consistent with the results showing effectiveness of COX-2 inhibitors in the reduction of the MDSC level through blocking COX-2 and subsequent inhibition of the STAT3 pathway (43, 44, 49, 50). Another arachidonic acid metabolite, leukotriene B4 (LTB4), a product of 5-lipoxygenase (5LO), acts as a chemoattractant for MDSCs, leading to their accumulation. Deficiency of 5LO is associated not only with a lowered circulation level of MDSCs but also with decreased activity of ARG1 and iNOS (51).

The tumor microenvironment stimulates MDSCs also by other factors induced by local hypoxia and low pH (52, 53). One of them is hypoxia-inducible factor (HIF). Over-expression of HIF-1α and also HIF-2α is associated with poor prognosis in the majority of cancers, including CRC (54). HIF-1α is associated with increased activity of ARG1 and iNOS in MDSCs, leading to stronger inhibition of T-cell functions (55). Moreover, HIF-1α can also enhance the suppressive nature of MDSCs by inducing expression of programmed death-ligand 1 (PD-L1) (56), a ligand for PD-1, leading to inhibition of IL-2 production and decreased proliferation of cytotoxic T cells (56, 57). Additionally, HIF-1α, by binding to a conserved hypoxia response element in the *V-domain of Ig suppressor of T-cell activation* (VISTA*)* promoter, upregulates VISTA expression on MDSCs, thereby inducing their suppressive activity in the tumor microenvironment (58).

Many studies have shown that not only soluble mediators but also extracellular vesicles, e.g., exosomes secreted by tumor cells, may directly induce MDSC development and modulate their activity (59). This was demonstrated for many malignancies, including melanoma, breast, lung, and CRC (60). The role of cancer exosomes in CRC is complex, based on the type of cargo material transferred from cancer cells to the cells of the immune system, including MDSCs. This may occur through the delivery of tumor proteins, e.g., FasL (61) and Hsp72 (62), mRNA (63), and non-coding microRNAs (miRNA) (64). The role of miRNA in CRC, in particular, has been documented recently, with an elevated level of miRNA-21 in patients' sera correlating with poor prognosis (65, 66).

### MDSC Action in CRC

The suppressive function of MDSCs in CRC is mainly associated with their ability to inhibit T-cell proliferation and to stimulate Treg development (15). One of the important factors involved in interactions between T cells and MDSCs is L-arginine, an amino acid that is essential for T-cell proliferation and proper functioning. MDSCs highly express ARG1, which uses L-arginine, causing its depletion from the microenvironment (21, 22), which in turn affects T-cell functionality. Lack of L-arginine blocks T-cell proliferation and decreases expression of CD3ζ chain and IFNγ production (67–69). Studies on CRC have shown that MDSCs impair T-cell activation through O${}_{2}^{-}$ production and iNOS activity (70, 71), which can be reversed by MDSC depletion or the use of iNOS and O${}_{2}^{-}$ inhibitors (72). The mechanism of ARG1- and iNOS-dependent T-cell suppression has been explained by studies showing that, under conditions where the L-arginine level is reduced due to ARG1 activity, L-arginine is preferentially used by iNOS for O${}_{2}^{-}$ and NO production, while under normal conditions, where the L-arginine level is high, only NO is produced (73). After mutual reaction of NO with O${}_{2}^{-}$, a strongly reactive oxidizing agent, peroxynitrite (ONOO^−^), is formed. It can cause nitration of proteins (74, 75) as well as the induction of T-cell apoptosis through the TCR/CD3 complex tyrosine phosphorylation pathway (22, 76, 77). Recent results have also shown that MDSC level correlates with reduction in the adaptive immune response to tumor antigens, e.g., MUC-1, both by lowering the production of specific antibodies and activation of tumor-specific T cells (27).

The interactions between MDSCs and Tregs in cancer are well-documented. Mainly, the activation of Tregs by MDSCs is caused by cytokines, including IL-10 and TGF-β, where the latter is also associated with MDSC induction (78). However, the relationship between MDSCs and Tregs in CRC is questionable. Some authors indicate that MDSCs in CRC do not induce Tregs development *in vitro* (70). On the other hand, mouse MDSCs were able to induce Tregs *in vitro* and *in vivo* through the IL-10- and IFN-γ-dependent pathway (79).

In addition to the role of MDSCs in immunosuppression that is observed during tumor progression, they may also directly stimulate tumor growth and metastases, inducing, in cooperation with VEGF, angiogenesis. Furthermore, MDSCs may introduce high levels of MMP9 and pro-MMP9 into the extracellular milieu, regulating VEGF bioavailability for colorectal cancer cells (80, 81). At the initial stage of cancer, MDSCs, through TGF-β, can also induce the epithelial to mesenchymal cell transition (EMT) process, which is essential for metastases at the late stage. These cells participate in extracellular matrix degradation in order to prepare distant tissue for receiving metastatic cells (82, 83). The latest findings reveal that PMN-MDSCs also enhance CRC growth by exosomes and exosomal protein S100A9 in the tumor microenvironment, especially under hypoxic conditions (84).

**Figure 1:**
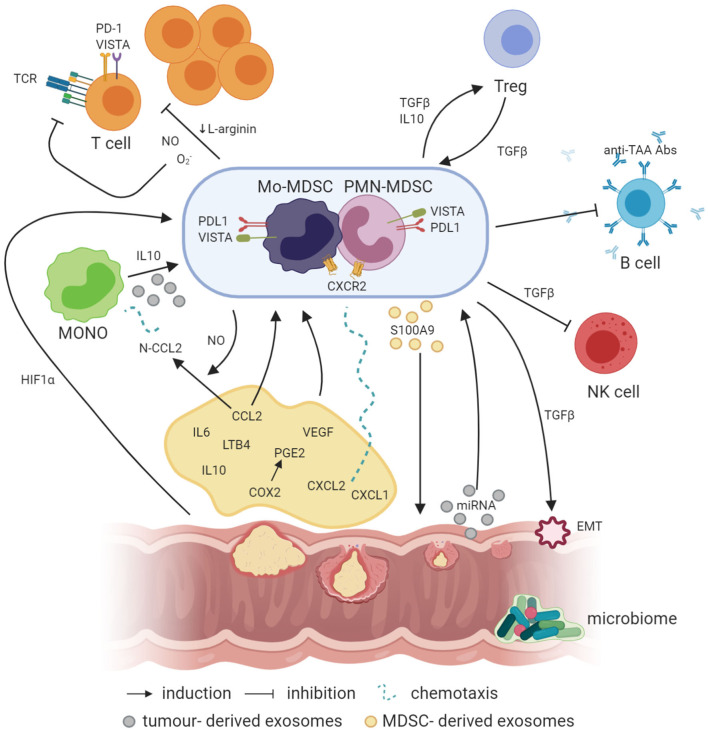
Crosstalk between MDSCs and other cells in the cancer microenvironment (created with BioRender.com). Factors like PGE2, IL-6, IL-10, and LTB4 are involved in the induction of MDSCs, where IL-10 can also be involved in the generation of Mo-MDSCs from circulating blood monocytes. In addition, NO produced by iNOS is required for the production of N-CCL2 from CCL2, acting as a chemoattractant for monocytes. In a similar manner, CXCL1 and CXCL2 binding to CXCR2 may recruit MDSCs to the tumor bed. Simultaneously, exosomes containing exosomal S100A9 protein are released by PMN-MDSCs, supporting the tumor growth. On the other hand, EVs generated by the tumor transfer biologically active tumor-related factors, e.g., proteins and miRNAs, which may also be involved in the induction of MDSCs from infiltrating monocytes. Moreover, hypoxia *per se* and hypoxia-related factors, including HIF1a, are also responsible for the induction of the expression of suppressive molecules such as VISTA or PD-L1 on the surface of MDSCs, which act through VISTA receptor and PD-1 on the T cells, respectively. TGFβ produced by MDSCs has a number of suppressive actions, e.g., MDSCs, through TGFβ, can induce the epithelial to mesenchymal cell transition (EMT) process, which is essential for metastasis formation, or inhibit NK cells. Moreover, TGFβ has a great influence, together with IL-10, on the induction of Tregs, while Tregs, producing TGFβ, induce in return MDSCs as a result of a positive feedback loop. In addition, MDSCs may also inhibit the production of antibodies and T cells directed against tumor-associated antigens (TAA), such as MUC1. Additionally, NO, O2-, and a reduced concentration of L-arginine, which are associated with MDSC activity in the tumor microenvironment, inhibit T-cell proliferation. Moreover, NO by itself can modify TCR structure and induce T-cell apoptosis.

Both populations of MDSCs can effectively inhibit T-cell activity but using different mechanisms (85, 86). Some authors suggest that Mo-MDSCs are more suppressive than PMN-MDSCs (87), while others show the opposite result (88, 89). PMN-MDSCs are mainly responsible for ROS production, while Mo-MDSCs have high expression of iNOS, producing large amounts of NO, which has a longer activity than ROS. Thus, PMN-MDSCs, in contrast to Mo-MDSCs, need direct cell-to-cell contact to suppress T cells (85, 90). In this context, it has been documented that PMN-MDSCs preferentially settle the peripheral lymphoid organs, while Mo-MDSCs mainly persist in the tumor bed (85). In addition, MDSCs can also downregulate innate immune response, e.g., affecting the activity of NK cells (91). The crosstalk between MDSCs and cells in the CRC microenvironment is summarized in Figure 1. According to some authors, in human CRC, a major proportion of the MDSCs in peripheral blood are PMN-MDSCs (86). However, there are also studies showing an increased level of both populations (92–95). Additionally, an e-MDSC population was also detected in CRC patients (27, 96).

## Detection of MDSCs in CRC

The composition of phenotype markers used for MDSC detection and characterization in CRC quite often differs between studies. The phenotype markers and functional characteristics of MDSCs from various studies on human CRC are presented in Table 1. While the majority of the authors agree that the general phenotype of MDSCs is CD11b^+^ HLA-DR^−^ Lin^−^ CD33^+^ or functional markers, e.g., iNOS^+^ and ARG1^+^, there is no consensus with respect to more specific markers such as CD14, CD15, PD-L1, or CD124 (IL-4αR). The recent recommendations of the COST-Mye-EUNITER consortium provide the minimal phenotype characteristics necessary to identify cells as MDSCs: CD14^−^CD11b^+^CD15^+^(or CD66b^+^) for PMN-MDSCs; CD11b^+^CD14^+^HLA-DR^low/−^ CD15^−^ for Mo-MDSCs, and Lin^−^(CD3/14/15/19/56)/HLA-DR^−^/CD33^+^ for e-MDSCs (17).

**Table 1:** The phenotype markers and functional characteristics of MDSCs as published in various studies on human CRC.

**Orgin/Tumor stage**	**Phenotype**	**Suppressive activity**	**References**
Circulating/I-IV	Lin^−^ HLA-DR^−^ CD11b^+^ CD33^+^ CD13^+^ CD115^low^ CD117^low^ CD124^low^ CD14^−^ CD15^−^ CD66b^−^ CD34^−^ CD39^+^ CD73^−^ PD-L1^low^ PD-L2^−^ PD-1^−^	MDSCs correlate with tumor metastasis. Inhibition of CFSE-labeled autologous CD3+ T cell proliferation at 2:1 ratios with MDSCs in the absence or presence of CD3/CD28 antibody stimulation for 3 days.	(26)
CD33+ from PBMC were co-cultured with SW480/SW620 cells to induce tumor MDSCs	CD33+ CD11b+ HLA-DR^−^, CD14^+^ CXCR4^+^ CD39^+^ ARG-1^+^ iNOS^+^ ROS^+^ PD-L1^+^ CD73^−^ CD117^+/−^ CD34^+/−^ CD66b^+/−^ CD15^weak^	Tumor-induced MDSCs promoted SW480 and SW620 cell growth in a co-culture system *in vitro*. Tumor-induced MDSCs suppressed the proliferation of PBMCs labeled with CFSE more strongly than CD33^+^ cells cultured in medium alone.	(70)
Circulating/tumor tissue	CD33^+^ CD11b^+^ HLA-DR^−^ CD14^+^ CXCR4^+/−^ CD39^+/−^ ARG-1^+^ iNOS^+^ PD-L1^+^ ROS^+^ CD73^−^ CD117^+/−^ CD34^+/−^ CD66b^+/−^ CD15^weak^ MDSCs from tumor tissue have higher PD-L1 expression	Advanced disease stage was associated with an elevated level of circulating MDSCs; also, tumor resection reduces the level of circulating MDSCs and Tregs measured 7 days after surgery.	
Circulating/IV	CD14+ HLA-DR^−/low^ S100A9^high^ iNOS^+^	–	(71)
Circulating/tumor tissue/III IV	CD124^+^CD14^+^ CD124^+^CD15^+^ tumor tissue CD15^+^ CD14^+^	Mixed lymphocyte reactions in which gamma-irradiated PBMC, CD14^+^, CD14^−^, and PMN from CRC patients were added as stimulator to responder PBMC derived from healthy donors. These experiments showed two main subpopulations with suppressive activity present among CD14^+^ monocytes in one and among PMN in the other.	(93)
Colorectal tumor/III	PMN-MDSCs CD45^+^ Lin^−^ HLA-DR^−^ CD11b^+^ CD33^+^ CD66b^+^ Mo-MDSCs CD45^+^ Lin^−^ HLA-DR^−^ CD11b^+^ CD33^+^ CD14^+^	PMN-MDSCs isolated from tumor inhibited the proliferation of activated autologous CFSE-labeled T cells and IFN-γ production in medium containing CD3 and CD28.	(94)
Circulating	CD33^+^ HLA-DR^−^ CD11b^+^ CD15^+^ CD33^+^HLA-DR^−^CD11b^+^CD15^−^ CD33^+^HLA-DR^−/*low*^CD14^+^	Upregulated plasma levels of IL-6 and IL-10, where IL-6 correlates with 15^+^ MDSCs and IL-10 with 15^−^ MDSCs. Also, CD15^+^ and CD15^−^ MDSCs correlated with reduced IFN-***α*** responsiveness in CD4^+^ T cells.	(95)
Circulating/Metastasis	PMN-MDSCs CD33^+^ HLA-DR^−/low^ CD15^+^ CD124^+^ PD-L1^+^ CD73^+^ CD39^+^ Mo-MDSCs CD33^+^ HLA-DR^−/low−^ CD14^+^ PD-L1^+^ CD73^+^ CD39^+^	Accumulation of PMN-MDSCs was associated with poor prognosis; also, PMN-MDSCs have higher levels of PD-L1, CD39, and CD73 expression and a stronger immunosuppressive function than Mo-MDSCs. Reduced TNF-α production and Ki67 proliferation marker of CD3^+^ T cells, especially by PMN-MDSCs.	(89)
Circulating/I-IV	CD33^+^ CD11b^+^ HLA-DR^−/low^ CD15^−^CD14^+^ ARG-1^+^ CD33^+^ CD11b^+^ HLA-DR^−^ CD15^+^ CD14^−^ARG-1^++^	–	(96)
Tumor tissue/I-IV	CD33^+^ CD11b^+^ HLA-DR^−/low^ CD15^−^CD14^+^ ARG-1^+^ CD33^+^ CD11b^+^ HLA-DR^−^ CD15^+^ CD14^−^ARG-1^+^ CD33^+^ CD11b^+^ HLA-DR^−^ CD15^−^ CD14^−^	–	
Circulating	PMN-MDSCs CD14^−^CD33^+^HLA-DR^−^CD66b^+^	Human MDSCs increase fatty acid uptake and expression of FAO-related enzymes, and, in mice, inhibition of FAO blocked the tolerogenic function and immunosuppressive mechanisms of MDSCs. Inhibition of CFSE-labeled CD3^+^ T-cell proliferation after co-culturing with MDSCs from mice in the presence of anti-CD3.	(86)
Circulating	Mo-MDSCs CD14^+^HLA-DR^−/lo^ PMN-MDSCs CD33^+^ CD11b^+^ CD14^−^ CD15^+^ SSC^hi^	Mo-MDSC population was significantly expanded in CRC patients; the immunosuppressive capacity of these cells was evaluated in a T-cell suppression assay using a 3-way allogenic mixed leukocyte reaction (MLR).	(92)
Circulating/cancer and adenoma	Total MDSCs: CD11b^+^HLA-DR^−/low^ CD33^+^ PMN-MDSCs: CD11b^+^HLA-DR^−/low^ CD33^+^ CD15^+^ CD14^−^ Mo-MDSCs: CD11b^+^HLA-DR^−/low^ CD33^+^ CD15^−^ CD14^+^ e-MDSCs: CD11b^+^HLA-DR^−/low^ CD33^+^ CD14^−^ CD15^−^	PMN-MDSCs are the main immunosuppressive population, as depletion of CD15^+^ cells spares Mo-MDSCs and eliminates most of the suppression of T-cell proliferation and interferon production. MDSC levels negatively correlated with anti-MUC1 IgG levels.	(27)

## Targeting MDSCs in CRC

Despite the availability of chemo- and immunotherapy, surgery is still the primary method of CRC treatment. However, in a mouse model, it was shown that surgical removal of tumor mass recruits MDSCs to the peritoneal cavity and promotes tumor progression due to the surgical trauma, downregulating the CXCL4 expression. CXCL4 inhibits tumor growth and angiogenesis, which might be due to its inhibitive impact on the recruitment of MDSCs (97). In this context, it seems that MDSC-targeted therapy is urgently required for this type of cancer.

There are numerous studies concerning different small-molecule compounds that are able to inhibit the suppressive activity of MDSCs. In this section, however, the compounds with potential for CRC treatment are mainly being discussed. One such is AT38, an inhibitor of RNS, which was used in a mouse model of CRC where it proved to effectively reduce nitration of chemokines, including CCL2. Administration of AT38 also decreased the level of iNOS and ARG1 (35). Another example is nitroaspirine, which, in a mouse model, increased the number of tumor antigen-specific T cells and reduced both ARG1 and iNOS activity in MDSCs (98). Triterpenoids were also shown to reduce the suppressive functions of MDSCs through downregulation of ROS and inhibition of STAT3. However, they did not exert any effects on ARG1 activity, on NO production, or on the frequency of MDSCs (99). In the human CRC, amiloride, normally used to reduce high blood pressure, can also inhibit tumor exosome formation, which has been shown to induce suppressive functions in MDSCs (62). It was also reported that H_2_ blockers, e.g., cimetidine, appear to induce apoptosis of MDSCs through a Fas-FasL-dependent pathway (100).

Another therapeutic approach involves the reduction of MDSC expansion by using COX2 or PGE2 inhibitors, as PGE2 production could be associated with MDSC expansion in cancer (43). Such inhibitors, e.g., indomethacin, celocoxib, melocoxib, and acethylosalicylo acid (ASA), were able to reduce tumor growth in various tumor models, including CRC (101–103). This treatment could also modulate MDSC functions by inhibiting ARG1 expression and ROS and NO production (104, 105). ASA also reduced the level of chemokines, including CCL2, a potent chemoattractant for MDSCs (106). Another way to block MDSC accumulation is the inhibition of stem cell factor (SCF), which causes MDSC recruitment when produced in the tumor environment (107).

Another option for targeting MDSCs is inducing their differentiation. For example, curcumin used in a mouse model of CRC was able to decrease the level of PMN-MDSCs and to induce differentiation of Mo-MDSCs into cells with M1-like phenotype (108).

Another strategy for potential MDSC-targeted therapy was suggested by Condamine et al. who pointed to a shorter lifespan for MDSCs compared with neutrophils and monocytes (109). This was associated with their increased apoptosis rate in the periphery, related to high expression of *TNF-related apoptosis–induced ligand* receptors (TRAIL-Rs) due to the stress in endoplasmic reticulum (ER) occurring under pathophysiological conditions like cancer. Thus, targeting TRAIL-Rs by selective agonists can be considered as a future therapy for reducing MDSC activity and number (109).

Immunotherapy designed to target the checkpoint inhibitors of the PD-1–PD-L1 pathway is currently one of the most promising possibilities for reducing MDSC activity. Currently, four monoclonal antibodies are already approved by the FDA for the inhibition of this pathway: anti-PD-1 nivolumab and pembrolizumab, and anti-PD-L1 atezolizumab and avelumab. These inhibitors and several other checkpoint modulators are under clinical investigation for CRC treatment (110). In the clinical studies, nivolumab and pembrolizumab showed good response rates of 26 and 57%, respectively (111). Better results were obtained in the case of nivolumab combined with ipilimumab (anti-CTLA-4) (111–113). However, in the context of MDSCs, more satisfactory results were obtained where the PD-L1 inhibitor was used (56). Recently, several chemotherapeutic agents, e.g., gemcitabine, 5-fluorouracil, and doxorubicin, which are used in conventional cancer chemotherapy have been found to reduce MDSC numbers through the induction of apoptosis in tumor tissues as well as in the peripheral lymphoid organs (114–116), and combining these agents with immunotherapy improved survival of tumor-bearing hosts. In keeping with this, Limagne et al. in their study, provided a clinical rationale for combining chemotherapy with anti-PD-1/PD-L1 antibodies for more effective reduction of the immunosuppression caused by PMN-MDSCs in metastatic CRC (89). In this context, FOLFOX (5-fluorouracil + oxaliplatin) chemotherapy was shown to act synergistically with anti-PD-1 (117).

In the context of immunotherapy, it is worth mentioning the heterogenic genetic composition of CRC, which has important therapeutic implications. The effectiveness of immunotherapy, particularly immune checkpoint inhibition therapy, such as CTLA-4 and PD-1, has been confirmed in mismatch-repair-deficient (dMMR) and microsatellite instability-high (MSI-H) (dMMR-MSI-H) tumors, while it was ineffective in mismatch-repair-proficient (pMMR) and microsatellite instability-low (MSI-L) (pMMR-MSI-L) tumors (118). This resistance for immunotherapy of MMR-MSI-L tumors results from the inability of immune cells to recognize MSI-L mutated tumor cells and thereby reduced T-cell infiltration (119). However, it was noticed that pMMR-MSI-L tumors are more extensively infiltrated by Tregs and MDSCs than dMMR-MSI-H, which may also explain the poor immune response (120). Thus, to use of MDSC-targeted therapy seems to be a beneficial opportunity to assist the effectiveness of surgery in patients with pMMR-MSI-L cancer.

## Conclusions

Tumor develops a variety of mechanisms to escape from immune system surveillance, including the generation of MDSCs. There is substantial evidence that MDSCs are involved in CRC development and progression. MDSCs can be detected both in the peripheral blood and tumor tissue; however, it is not known if both or one of them are relevant for predicting the prognosis for patients in the clinic. Therefore, more in-depth investigation of the mechanisms of MDSC actions in the tumor bed is still needed. Finally, more advanced pharmacological data on specific treatments targeting MDSCs are required. This could significantly improve the effectiveness of the treatment of CRC patients, and also those with pMMR-MSI-L tumors, who respond poorly to current forms of immunotherapy.

## Author Contributions

IS wrote the paper. JB critically revised the paper. All authors contributed to the article and approved the submitted version.

## Conflict of Interest

The authors declare that the research was conducted in the absence of any commercial or financial relationships that could be construed as a potential conflict of interest.
